# Mitochondrial Exhaustion of Memory CD4 T-Cells in Treated HIV-1 Infection

**DOI:** 10.20900/immunometab20220013

**Published:** 2022-04-28

**Authors:** Souheil-Antoine Younes

**Affiliations:** Department of Pathology, Pathology Advanced Translational Research (PATRU), School of Medicine, Emory University, Atlanta 30322, USA

**Keywords:** mitochondria, exhaustion, CD4 T-cells, pgc1α

## Abstract

People living with HIV (PLWH) who are immune non-responders (INR) to therapy are unable to restore their CD4 T-cell count and remain at great risk of morbidity and mortality. Here the mitochondrial defects that characterize memory CD4 T-cells in INR and causes of this mitochondrial exhaustion are reviewed. This review also describes the various reagents used to induce the expression of the peroxisome proliferator-activated receptor gamma coactivator 1-alpha (PGC1α), the master regulator of mitochondrial biogenesis, which can restore mitochondria fitness and CD4 T-cell proliferation in INR. Due to sustained heightened inflammation in INR, the mitochondrial network is unable to be rejuvenated and requires attenuation of mediators of inflammation to rescue mitochondria and CD4 T-cell counts in INR.

## INTRODUCTION

Despite effective control of HIV replication with antiretroviral therapy (ART), a proportion of ART-treated persons are unable to reconstitute CD4 T-cell counts to levels observed in uninfected individuals [1,2]. These immune non-responders (INR) are at greater risk of morbidity and mortality than are immune responders (IR) in whom CD4 T-cell counts are restored [3,4]. Bacterial translocation from the gut to the bloodstream is a hallmark of HIV infection that leads to systemic immune activation [5] and INR plasma level of bacterial translocation markers are elevated [1,6]. As reported by our group [7], INR are characterized by memory CD4 T-cell mitochondrial dysfunction and spontaneous death of cycling (Ki67^+^/CD71^+^) memory CD4 T-cells in vitro. We also validated mitochondrial dysfunction of CD4 T-cells by electron microscopy imaging (EMI) and by metabolic analysis [8]. The cause of this mitochondrial dysfunction is attributed to heightened levels of inflammatory mediators that prevents CD4 T-cell reconstitution by impeding mitochondrial biogenesis discussed in the following section.

## INFLAMMATION IMPEDES MITOCHONDRIAL NETWORK AND MEMORY CD4 T-CELL MAINTENANCE

Following viral or bacterial infection, naive T-cells get activated and expanded robustly to generate effector cells that clear the infection. Effector cells undergo a contraction phase and a small population of memory T-cells persist and are responsible for the long-term immune memory and protection [9]. Effector T-cells are metabolically active, with high rate of glycolysis and oxidative phosphorylation (OXPHOS) essential to their highly proliferative state [10]. Memory T cells however rewire to a more quiescent metabolism and rely mainly on fatty acid oxidation (FAO) and OXPHOS metabolic pathways [11]. A common feature of memory T-cells during immune responses is the ability to respond rapidly and efficiently to a previously encountered antigen, which results in rapid control of infections [12]. In chronic infections and in inflammatory conditions memory T-cells become exhausted as further discussed in this review.

Chronic immune inflammation is a hallmark of HIV-1 infection that persists years after successful ART [13]. Inflammation induces aerobic glycolysis (Warburg effect) which provides a quick energy source and rapid access for processes linked to cellular proliferation [14]. Antigen persistence and inflammation induce exhausted T-cells that upregulate immune checkpoint inhibitors such as programmed cell death protein 1 (PD-1) [15,16]. PD-1 ligation attenuates phosphoinositide 3-kinase (PI3K), Akt (Protein Kinase B), and mammalian target of rapamycin (mTOR) signaling leading to the inhibition of the T-cell receptor signaling, cell cycle arrest, and cell exhaustion [17]. It has been shown recently that type I interferons, essential for host defense against viruses [18,19], are major inducers of checkpoint inhibitors on T-cells including PD-1 [18]. Using the mouse chronic lymphocytic choriomeningitis virus (LCMV clone 13), it was shown that PD-1 expression regulated early glycolytic and mitochondrial alterations and repressed PGC-1α [20], essential for mitochondrial biogenesis. Prior and post ART intervention, INR patients have the highest levels of PD-1 expression in memory CD4 T-cells [21,22].

## INFLAMMATION PREVENTS REGULATORY CD4 T-CELL DEVELOPMENT

Regulatory CD4 T-cells (Tregs) reduce inflammation and are essential components in the regulation of T-cell homeostasis by the expression of the transcriptional factor foxp3 and the production of the transforming growth factor β (TGF-β) [23–25]. Tregs are produced in the thymus and in the periphery mainly in gut mesenteric lymph nodes (MLN) [26]. The thymus of PLWH is dysfunctional [27,28] which renders the MLN the main source of Treg generation detected in peripheral blood. We have performed transcriptomic analysis on cycling-proliferating (CD71^+^) and non-cycling (CD71^−^) CD45RO^+^ CD4 T-cells from healthy participants, IR, and INR [7]. We have found downregulations of genes implicated in Treg signature, in Foxp3 target genes essential for Treg function, in TGF-β-signaling, and in OXPHOS. INF-α response genes, however, were significantly upregulated in CD71^+^ memory CD4 T-cell population in which ~50% of the cells are Tregs [7]. Genes implicated in mitochondria function such as mitochondrial transcriptional factor A (mTFA) and PGC-1α were also downregulated in CD71^+^ Tregs in INR patients [7]. Although we have focused our study on Tregs and cycling Tregs, the defects described above were also found in memory CD4 T-cells of INR (SAY, unpublished data). The study of Zhao et al. [29] have confirmed our finding in a separate INR cohort as the study focused on total memory CD4 T-cells. In addition, the mitochondrial defect was validated by seahorse assay that showed lower oxygen consumption rate (OCR) in CD4 T-cells from PLWH in general compared to healthy controls while INR have the lowest basal and after-stimulation OCR among the studied groups [29,30].

## LYMPHOPENIA AND TREGS DEPLETION

It is well documented that during the initial phase of HIV-1 infection there is a massive depletion of gut-resident CD4 T-cells in HIV-1 [31] and SIV [32] infections. Although reports claimed that Tregs are spared from HIV-1 depletion [33], Treg cells are highly proliferative in vivo ([34–36] and potentially highly susceptible to HIV-1 depletion. The finding [33] claiming that high frequencies of Treg persisted during the initial phase of HIV-1 infection may be due to the methods used to identify Tregs which are based on Foxp3 and CD25 expression both induced as well following CD4 T-cell activation [37–39]. In mouse model, naïve CD4 T-cells transferred into the lymphopenic Recombination Activating gene 1 Knock-out (Rag-KO) mice massively proliferated and caused colitis in the transferred mice. When Tregs were transferred with naïve CD4 T-cells colitis did not occur [40]. By analogy, in HIV-1 infection the massive depletion of proliferative cells such as Tregs would render CD4 T-cell proliferation out of control. Indeed, it was reported that memory CD4 T-cells in INR have the highest levels of Ki67/CD71^+^ among PLWH [1,2], yet sorted CD71^+^CD4 T-cells from INR were unable to divide in vitro and displayed gene signatures associated to apoptosis [7]. As discussed above and elsewhere [41] inflammatory milieu characteristic of INR impedes Treg development.

## GUT BACTERIAL TRANSLOCATION AND ROLE IN THE HEIGHTENED INFLAMMATION IN INR

Emerging evidence highlight the role of gut microbiota in modulating Immune cells [42,43]. PLWH have gut microbiome flora containing more pro-inflammatory bacteria than individuals without HIV infection [44–46]. It was suggested that the shift in the microbiome contributed to the heightened levels of microbial translocation from the gut to the bloodstream, and this was associated with systemic inflammation and immune activation that characterize all stages of HIV disease [44–46]. Elevated levels of bacterial *Serratia* genera detected by DNA/RNA deep-sequencing technology (PathSeq) was found in plasma from INR demonstrating that the translocation of *Serratia* to the bloodstream was accompanied by elevated levels of inflammatory plasma cytokines (IL-1, IL-6, and IL-8) possibly as a consequence of monocytes and macrophage activation by *Serratia* products [47]. Thus, bacterial translocation contributes to the heightened inflammation detected in INR.

## GUT-BACTERIAL DERIVED SOLUTES AND ROLE IN MITOCHONDRIAL DYSFUNCTION OF CD4-T CELLS IN INR

**Figure 1.**
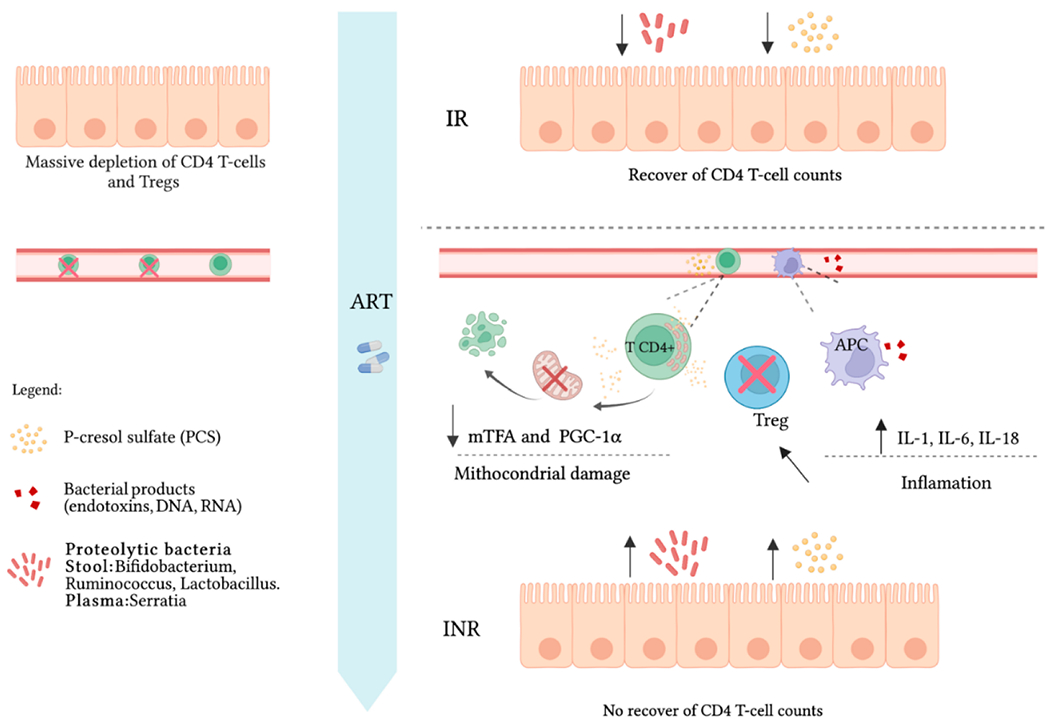
Following HIV-1 infection, a massive depletion of gut-CD4 T and Tregs occurs in INR; following ART, the heightened levels of proteolytic bacteria producing toxins (e.g., PCS) impedes CD4 T-cell reconstitution and promotes CD4 T-cell exhaustion. Bacterial translocation to the bloodstream (e.g., *Serratia*) induces inflammatory milieu that impedes Treg differentiation and function rendering CD4 T-cell proliferation out of control. CD4 T-cells become exhausted and loose the capacity to regenerate mitochondria by diminishing the expression of mTFA and PGC1α both essential for mitochondrial biogenesis.

Gut-bacterial derived solutes (GBDS) such as P-cresol sulfate (PCS) and Indoxyl-sulfate (IS) are the most studied so called “uremic toxins” that accumulated in the plasma of patients with chronic kidney disease (CKD) and are associated with cardiovascular and kidney disease [48–50]. PCS and IS are generated following protein degradation by the proteolytic gut-bacterial flora [51]. We have found significant enrichment of PCS and IS concentrations in the INR plasma of multiple cohorts, and CD4 T-cell counts were negatively associated with plasma concentrations of PCS and IS [8]. In vitro, incubation of CD4 T-cells with PCS or IS from healthy donors blocked cell proliferation, reduced the expression of mitochondrial proteins such as COXIV and mTFA and induced cell exhaustion [8]. Stool sample analysis of bacterial diversity revealed enrichment of bacterial species capable of PCS production in INR samples [8]. Figure 1 recapitulates the mechanisms implicated in CD4 T-cell exhaustion in INR patients.

## RESTORATION OF MITOCHONDRIAL DYSFUNCTION

Recent reports suggest that restoration of mitochondrial biogenesis rescues T lymphocytes from exhaustion. Correcting mitochondrial dysfunction has been shown to restore the activity of exhausted HBV-specific CD8 T cells in chronic human hepatitis B infection [52]. Exhausted HBV-specific CD8 T cells showed signs of mitochondrial alterations. Improvement of mitochondrial and antiviral CD8 functions was reached by the usage of antioxidants suggesting a central role for reactive oxygen species (ROS) in T cell exhaustion. The authors used antioxidants mitoquinone and the piperidine-nitroxide MitoTempo to attenuate ROS production and that resulted in better mitochondrial function and restoration of CD8 T cell function [52]. In mice infected with LCMV clone13, which generates persistent viral infection and continuous antigen exposure, CD8 T cells specific for the virus become exhausted and are unable to effectively clear the virus. Exhausted CD8 T cells were rescued upon overexpression of PGC1α, which restored mitochondrial and CD8 T cell function [20]. Similar approach was used for CD8 T cells that infiltrated tumors in which overexpression of PGC1α restored mitochondrial and CD8 T cell function [53]. We have shown that memory CD4-T cells of INR have low mitochondrial function and low PGC1α expression [7]; we also showed that interleukin 15 (IL-15) restored PGC1α and mitochondrial transcriptional factor A (mTFA) expression in exhausted CD4 T-cells in INR. In addition, the ablation of mTFA by CRISPR/Cas9 attenuated mitochondrial function as measured by Seahorse assay [29] suggesting that mTFA, which is under the control of PGC1α, is essential for mitochondrial DNA replication and function.

**Figure 2.**
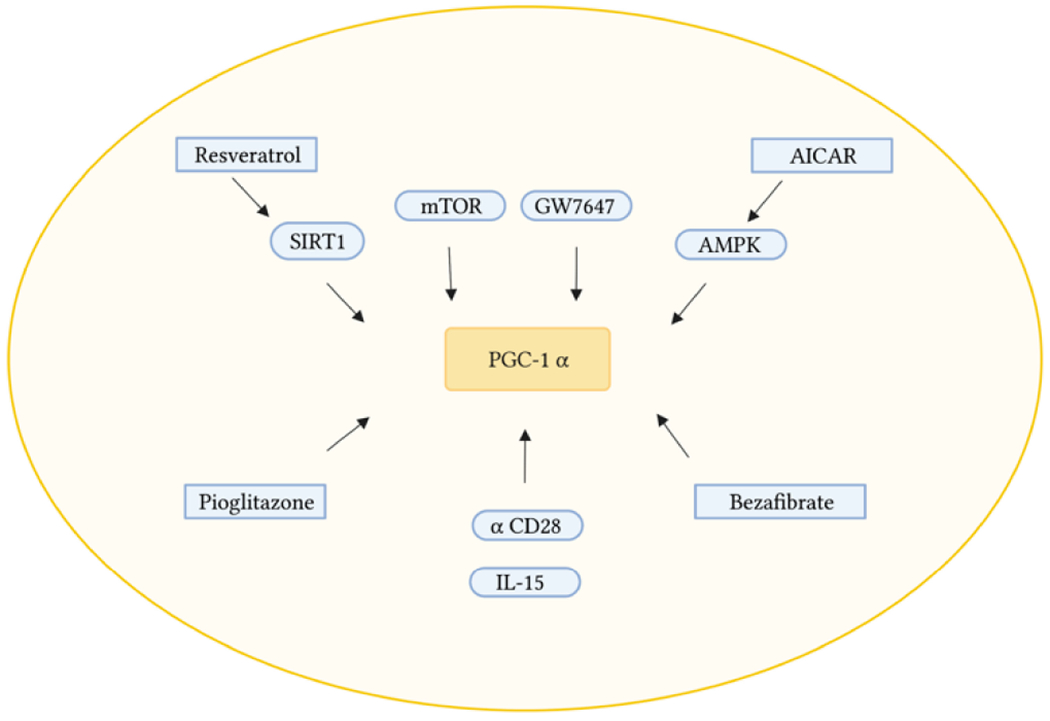
Overview of the possible induction of PGC1α-mediated mitochondrial biogenesis by resveratrol, bezafibrate, AICAR, GW7647, and Pioglitozone. Resveratrol: activator of SIRT1. AICAR: activate AMPK. Bezafibrate: PPAR pan-agonist. Pioglitazone and GW7647: PPARα and PPARγ agonists. IL-15: Activator of mTOR.

We have used several strategies to induce the induction of PGC1α, followed by monitoring CD4 T-cell proliferation in vitro upon stimulation with anti-CD3/CD28. We have employed the reagents described in Figure 2 to monitor PGC1α expression as well as cell proliferation rescuing.

During caloric excess PGC1α is in an inactive form [54]. In times of caloric restriction, energy becomes limited causing increases in the AMP/ATP ratio and NAD^+^/NADH ratio. The elevated levels of AMP and NAD^+^ have a direct impact on the activation of AMP kinase (AMPK) as binding of AMP (or ADP) facilitates phosphorylation of AMPK by upstream kinases. Activated AMPK induces the phosphorylation of PGC1α directly, or prime PGC1α for activation via deacetylation by Sirtuin 1 (SIRT1). This leads to mitochondrial proliferation and increased mitochondrial mass [54]. Resveratrol is an activator of SIRT1 [55]. 5-aminoimidazole-4-carboxamide ribonucleotide (AICAR), an AMP mimetic, can directly activate AMPK by phosphorylation of AMPK through upstream kinases [56]. Activation of AMPK causes phosphorylation of PGC1α, and SIRT1 activation via AMPK-induced increases in the NAD^+^/NADH ratio [56]. Bezafibrate is a peroxisome proliferator-activated receptor (PPAR) pan-agonist that induces the expression of PGC1α via the PPAR-responsive element in its promoter region [57,58]. GW7647 and pioglitozone are PPARα and PPARγ agonists respectively [58]. bezafibrate, GW7647, and pioglitozone were all being tested on CD4 T-cells and were able to induce foxp3 gene in regulatory CD4 T-cells [57] that rely on OXPHOS and mitochondria biogenesis for function [59–61]. Finally, we also used antioxidants mitoquinone and MitoTempo to address whether quenching ROS production may induce PGC1α and restore cell proliferation.

The reagents displayed above were neither able to induce PGC1α expression nor cell proliferation in CD4 T-cells from INR (SAY unpublished data). IL-15, however, was able to induce the expression of PGC1α via the activation of mTOR pathway in some but not all INR participants (SAY unpublished data). Indeed, it was shown that IL-15 induced mTOR activation [62–66] and the latter promoted PGC1α expression via the transcription factor yin-yang 1 (YY1) [67]. The data suggest that IL-15 induced PGC1α and rescued CD4 T-cell proliferation in CD4 T-cells, which were still able to rejuvenate their mitochondrial pool as discussed in the next section.

## IS THE MITOCHONDRIAL NETWORK EXHAUSTED IN INR CD4 T-CELLS?

Recent reports highlighted the impact of asymmetric mitochondrial distribution during cell division in which cells that received old mitochondria would be effector/short-lived compared to cells that had newly synthesized mitochondria which are endowed with stemness and memory ability [68–71]. In a recent report using human mammary epithelial cells (HMECs) as an in vitro model, old mitochondria supported OXPHOS whereas the electron transport chain of new mitochondria was immature with limited respiration capacity [71]. It was also shown that cells that inherited old mitochondria after mitosis displayed heightened OXPHOS and differentiated. Cells that inherited newly synthesized mitochondria had a higher pentose phosphate pathway activity. This promoted de novo purine biosynthesis and redox balance and was required to stemness and maintenance during early fate determination after cell division [71]. A similar mechanism of asymmetric mitochondrial distribution and long-term memory cells maintenance was described in CD8, CD4, and B lymphocytes [68,69]. Due to the inflammatory mediators exposed above (Figure 1), it is likely that INR CD4 T-cells are incapable of generating memory long lived CD4 T-cells due to the inability to asymmetrically distribute mitochondria in dividing cells. In fact, we have shown that sorted CD71^+^(Ki67^+^) memory CD4 T-cells from INR did not divide in vitro and had significant reduction of genes implicated in mitochondrial fitness [7].

## CONCLUSIONS

Strategies to reduce immune activation and inflammation have been employed in INR patients such as antibiotic treatment [72,73], fecal microbiota transplantation, or probiotic interventions [74,75] with mixed success [75]. Dietary approaches [76–78] however, appear to have a positive impact on lowering inflammation in INR. It is still unknown whether INR can generate CD4 T-cell compartment following the interventions exposed above. Attenuation of inflammation may be the key strategy to rejuvenate mitochondrial network and restore CD4 T-cell count in INR.
